# Diagnostic accuracy of digital breast tomosynthesis in combination with 2D mammography for the characterisation of mammographic abnormalities

**DOI:** 10.1038/s41598-020-77456-6

**Published:** 2020-11-26

**Authors:** Vithya Visalatchi Sanmugasiva, Marlina Tanty Ramli Hamid, Farhana Fadzli, Faizatul Izza Rozalli, Chai Hong Yeong, Nazimah Ab Mumin, Kartini Rahmat

**Affiliations:** 1 Department of Biomedical Imaging, University Malaya Research Imaging Centre, University of Malaya, 50603 Kuala Lumpur, Malaysia; 2 Department of Radiology, Faculty of Medicine, University Teknologi MARA, Sungai Buloh, Selangor Malaysia; 3 School of Medicine, Faculty of Health and Medical Sciences, Taylor’s University, 47500 Subang Jaya, Selangor Malaysia

**Keywords:** Oncology, Cancer

## Abstract

This study aims to assess the diagnostic accuracy of digital breast tomosynthesis in combination with full field digital mammography (DBT + FFDM) in the charaterisation of Breast Imaging-reporting and Data System (BI-RADS) category 3, 4 and 5 lesions. Retrospective cross-sectional study of 390 patients with BI-RADS 3, 4 and 5 mammography with available histopathology examination results were recruited from in a single center of a multi-ethnic Asian population. 2 readers independently reported the FFDM and DBT images and classified lesions detected (mass, calcifications, asymmetric density and architectural distortion) based on American College of Radiology-BI-RADS lexicon. Of the 390 patients recruited, 182 malignancies were reported. Positive predictive value (PPV) of cancer was 46.7%. The PPV in BI-RADS 4a, 4b, 4c and 5 were 6.0%, 38.3%, 68.9%, and 93.1%, respectively. Among all the cancers, 76% presented as masses, 4% as calcifications and 20% as asymmetry. An additional of 4% of cancers were detected on ultrasound. The sensitivity, specificity, PPV and NPV of mass lesions detected on DBT + FFDM were 93.8%, 85.1%, 88.8% and 91.5%, respectively. The PPV for calcification is 61.6% and asymmetry is 60.7%. 81.6% of cancer detected were invasive and 13.3% were in-situ type. Our study showed that DBT is proven to be an effective tool in the diagnosis and characterization of breast lesions and supports the current body of literature that states that integrating DBT to FFDM allows good characterization of breast lesions and accurate diagnosis of cancer.

## Introduction

Breast cancer is the most common cancer in women worldwide^1^. From 2009 to 2015, the 5-year relative survival rate of women with breast cancer was 99%, 85% and 27% for local disease, regional disease and breast cancer with distant metastases respectively^2^.

Mammography is a proven screening modality to reduce breast cancer mortality and effective in detecting early stage breast cancer. Digital breast tomosynthesis (DBT) is a technique in breast imaging that overcomes the problems of overlapping breast tissues in full field digital mammography (FFDM) by producing multiple cross­sectional images from reconstructed volume data. There has been a significant reduction in recall rates and an increase in breast cancer detection rates with the introduction of DBT into clinical practice^3,4^. Better visualization of lesions’ margin by DBT, particularly in dense breasts, has improved the sensitivity and specificity of lesion detection and allows for better categorization of suspicious and benign breast lesions^5,6^.

As DBT is a relatively new technique, limited research has been done on the diagnostic accuracy in the characterisation of lesions, particularly in multi-ethnic Asian population. Studies done in the Caucasian populations may not be reflective of other populations due to the different variables, such as ethnic differences, lifestyle and psychosocial factors^7^. Pathy et al. showed that 51% of Asian women were diagnosed with breast cancer before the age of 51 based on a hospital-based cancer registry from 1990 to 2007. This is in contrast to the Caucasian women, whereby only 23% of the total breast cancer incidence were younger than 50 year-old^8^. Asian women also presented with larger tumour size and presented at an advanced stage. 10% of patients in the Asian population has metastatic disease at initial presentation as opposed to 3–6% in the Western setting. The proportion of patients who were not treated, declined follow up or seeked traditional treatment were also not uncommon in Asian women^8^.

Therefore, this study aims to assess the diagnostic accuracy of DBT in combination with FFDM (DBT + FFDM) in the charaterisation of mammographic abnormality in opportunistic screening, targeted screening and diagnostic groups in a multiethnic Asian population.

## Methodology

This was a retrospective cross-sectional study involving consecutive 390 patients who had undergone DBT + FFDM imaging at the Department of Biomedical Imaging, University of Malaya Medical Centre (UMMC) between January 2015 and May 2017. The study was approved by Medical Ethics Committee of University Malaya Medical Centre (MECID No: 20154-1233).

### Patient selection

**Figure 1:**
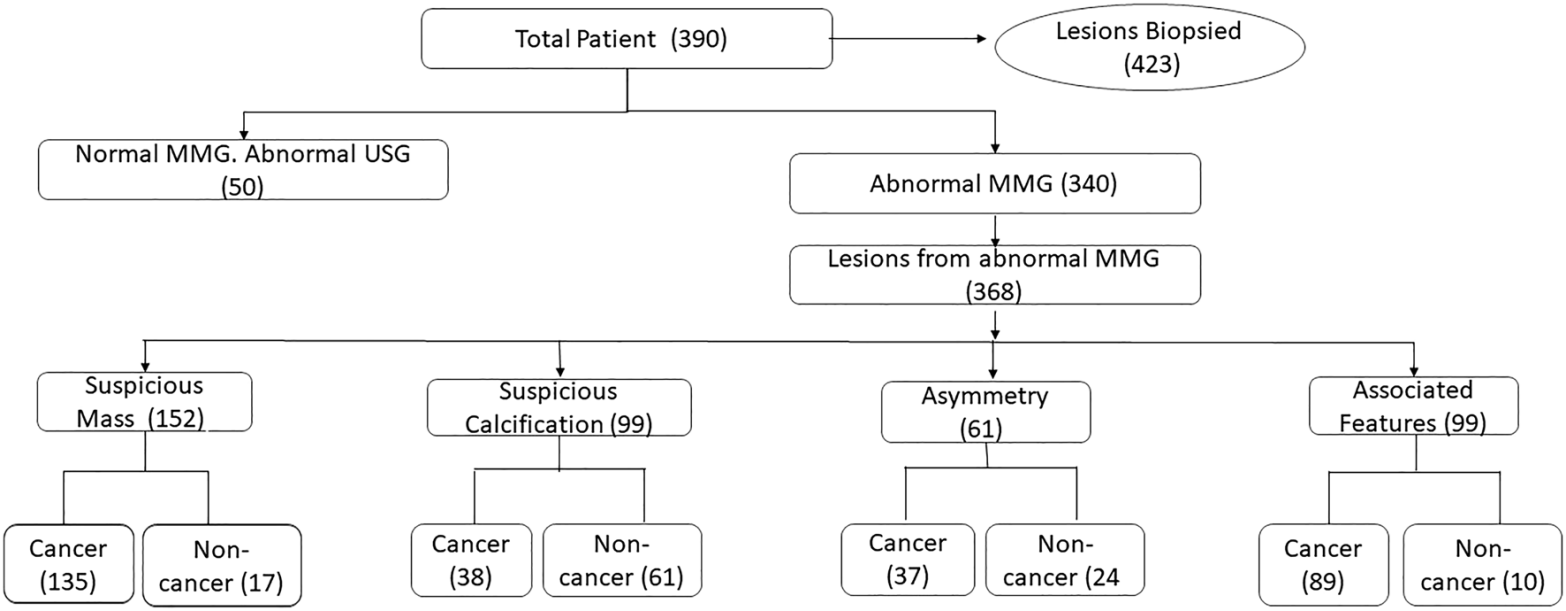
Flow chart of number of patients included in the study with number of lesions detected and final HPE results.

Patients who underwent DBT + FFDM with tissue diagnosis of the detected suspicious breast lesions were recruited. Inclusion criteria includes (1) patients presented for routine (opportunistic) screening (2) patients referred for triple diagnosis due to breast-related symptoms such as breast lumps, nipple discharge or breast pain, (3) symptomatic patients (diagnostic group), (4) women with high risk such as family history of breast cancer and on hormone replacement therapy (targeted screening group), (5) patients assigned to Breast Imaging Reporting and Data System (BI-RADS) 3, 4 or 5 with available histopathological diagnosis via core biopsy, excisional biopsy or mastectomy. Exclusion criteria were patients with known breast cancer, previous breast surgery or mastectomy, reconstructed breast and with breast implants. Flow chart of patients inclusion in the study with number of lesions detected and HPE results is summarized in Fig. 1.

### DBT + FFDM imaging

All patients underwent combined DBT + FFDM (Combo mode) using a 3D digital mammography system (Selenia Dimension, Hologic, Bedford, Massachusetts, USA) in the same setting. A 2D synthesized images were automatically produced by summing and filtering the tomosynthesis images using an image processing software (C-View software by Hologic). Hence, each patient had three sets of images, i.e. the conventional 2D FFDM, DBT and 2D synthesized images (C-View). All sets consist of CC and MLO views. The processed image data were sent from the acquisition workstation to the reading workstation.

All the patients were then subjected to supplementary breast and axillary ultrasound evaluation (Philips iU22; Philips Healthcare, Bothell, WA) with a high frequency (12.5 MHz) linear transducer.

### Data collection, storage and image assessment

All the acquired image viewing and analysis were done in SecurView DX workstation (Hologic, Bedford, Massachusetts, USA). Data of FFDM, DBT and C-View were stored in the hospital Picture Archiving and Communication System (PACS) (AWS PACS, Centricity, GE Healthcare, UK).

The FFDM, DBT and C-View images were analysed consecutively by two breast radiologists (MR and FF) of 7 and 5 years breast imaging experience in a research setting. The reporting and grading of the BI-RADS score were determined as per American College of Radiology (ACR)-BI-RADS 5th edition (2013) mammogram lexicon and the assessment criteria included breast density assessment, calcifications, mass, asymmetric density, architectural distortiona dn other associated features. The final assessment category of BI-RADS 3 (probably benign, likelihood of malignancy between 0 and 2%), BI-RADS 4 (suspicious of malignancy, likelihood of malignancy 2–95%) or BI-RADS 5 (highly suspicious of malignancy, likelihood of malignancy > 95%) was determined after an adjunct ultrasound evaluation as per the ACR BI-RADS classification. The ultrasound images were not included in the image assessment if they were performed. The readers were blinded of the ultrasound findings, histopathology report and patient’s outcome.

### Histopathological examination (HPE)

Biopsies for suspicious lesions were carried out either with stereotactic guided 14G core biopsy, 10G vacuum assisted stereotactic guided biopsy, ultrasound-guided core biopsy or freehand biopsy (for palpable lesions) by radiologists or clinicians who were not involved. n this study in a clinical setting. The biopsies were performed using 14G BARD Magnum instrument or 10G Mammotome Revolve Biopsy Device. HPE of breast specimens of patients who had undergone image-guided hook wire localization surgical excision and mastectomy were also included. Histopathology examinations were analysed and reported by histopathologists who were not involved in this study, in a clinical setting. Cancers were identified as invasive lobular, invasive ductal and ductal carcinoma in situ (DCIS). Cancers containing mixed invasive and in-situ components were classified as invasive.

### Statistical analysis

Statistical analysis was performed using Statistical Package for the Social Sciences (SPSS version 23.0) software. Breast lesions were characterized using ACR-BI-RADS 5th edition (2013) mammogram lexicon. BI-RADS category 3 was considered as benign lesion and BI-RADS category 4 and 5 were considered as suspicious for malignancy. HPE results were referred as the gold standard. Correlations between the quantitative and qualitative characteristic of the lesions with the HPE results were studied. The quantitative assessment includes the size of malignant lesions, while qualitative parameters include mass, calcification, asymmetry, architectural distortion and associated features. The sensitivity, specificity, PPV and NPV were determined for mass. PPV was calculated for calcification, assymetric density and other associated findings. PPV is calculated as (number of true positives)/(number of true positives + number of false positives), and NPV is (number of true negative)/(number of true negatives + number of false negatives).

Diagnostic accuracy of BI-RADS category were calculated and compared to the gold standard.

### Informed consent

Written consent was waived by the institution ethics committee due to retrospective nature of the study.

## Results

### Patients demography

A total of 387 women and 3 men were recruited in this study. Majority of the patients were Malay (39.7%), 33.1% were Chinese and 24.9% were Indian. The patients’ ages ranged between 28 and 86, with the majority in the 40–69 age group (79.8%, *n* = 331). 43.1% (*n* = 168) patients were in BI-RADS density C and D category. BI-RADS density category was not assigned for the 3 male patients. Table 1 shows the number of patients and HPE findings in diagnostic, opportunistic and targeted screening groups. There were 87.4% (*n* = 341) of the patients who did not have family history of breast cancer. Out of the 49 patients with family history of cancer (first or second degree relatives with breast cancer), 23 (46.9%) patients were positive for malignancy.

**Table 1:** Number of patients and HPE findings in each study group.

Group	*n* (%)	Benign *n* (%)	Malignant *n* (%)
Diagnostic	243/390 (62.3)	89/243 (36.6)	154/243 (63.4)
Opportunistic screening	110/390 (28.2)	91/110 (82.7)	19/100 (17.3)
Targeted screening	37/390 (9.5)	28/37 (75.7)	9/37 (24.3)

### HPE findings

There were 340 patients with 368 lesions detected on DBT + FFDM. 50 patients had unremarkable DBT + FFDM with 55 lesions identified on adjunct ultrasound. Hence, a total of 423 lesions were biopsied. There were 262 (61.9%) lesions with ultrasound-guided core biopsy performed, 47 (11.1%) lesions had stereotactic-guided core biopsy performed and 3 (0.7%) lesions had vacuum-assisted stereotactic-guided biopsy (VAB). Free hand biopsy accounted for 26.2% (n = 111) of the lesions.

Out of the 423 biopsied lesion, 196 (46.3%) were malignant and 227 (53.7%) were benign. The detailed HPE findings were outline in Table 2. The commonest benign lesion was fibrocystic disease and the commonest malignant lesion was invasive ductal carcinoma. There were 57% (n = 112) malignant lesions noted in the right breast and 35.5% (73) in the upper outer quadrant. No interval cancers detected in all patients diagnosed as benign after minimum of 2 years follow up.

**Table 2:** Histopathological findings of the lesions (*n* = 423).

Benign	*n* (%) 227/423 (53.7)	Malignant	*n* (%) 196/423 (46.3)
Fibrocystic disease	77/227 (34)	Invasive ductal carcinoma	157/196 (80.1)
Fibroadenoma/adenosis	56/227 (25)	Invasive lobular carcinoma	3/196 (1.5)
Usual ductal hyperplasia	15/227 (6)	Ductal carcinoma in situ	27/196 (13.8)
Benign Papilloma*	13/227 (6)	Others***	9/196 (4.6)
Others**	66/227(29)		

*Included in benign category all were confirmed benign papilloma in final surgical HPE.

**Other benign lesions include adipose tissue, fibroglandular tissue, mammary duct and acini and no malignancy.

***Other malignant lesions include neuroendocrine carcinoma, spindle cell carcinoma, solid papillary carcinoma with endocrine differentiation and diffuse large B-cell lymphoma.

Table 3 shows the distribution of malignant lesions in association with BI-RADS density. Out of the 144 cancers, 1 was a male patient hence BI-RADS density was not assigned. 65 cancers (45%) were detected in BI-RADS density B.

**Table 3:** Distribution of cancer in association with BI-RADS density.

BI-RADS density	Distribution % (*n* = 143)
A	23.08% (n = 33/143)
B	45.45% (n = 65/143)
C	25.87% (n = 37/143)
D	5.59% (n = 8/143)

For ethnicity, the percentage of patients diagnosed with cancer in each ethnic group were almost similar, with 71 (45.8%) Malays, 59 (45.7%) Chinese and 49 (50.5%) Indians. As expected, invasive ductal carcinoma was the most common cancer across all ethnicity and age groups.

There were 6.9% (n = 27 ) age < 40 years old, 79.7% (n = 311) age 40–69 and 13.3% (n = 52 )age ≥ 70 in the study population. Higher percentage of malignancy with increasing age group as depicted in Table 4. Table 4 is showing age group distribution of benign and malignant lesions.

**Table 4:** Distribution of benign and malignant lesions in different age groups.

Age group	% Benign	% Malignant
< 40	63% (n = 17/27)	37% (n = 10/27)*
40–69	57.6% (n = 179/331)	42.4% (n = 132/311)
≥ 70	23.1% (n = 12/52)	76.9% (n = 40/52)

*All malignant cases were age 30–39 in this group.

### Radiological features, BI-RADS category and HPE diagnosis

From DBT + FFDM diagnosis, 253 lesions were categorized as BI-RADS 4 and 132 lesions as BI-RADS 5. There were 37 lesions in BI-RADS category 3 biopsied as requested by the primary team. Table 5 summarises the association between BI-RADS category and HPE results. The PPV for BI-RADS 4a, 4b and 4c were 6%, 38.3% and 68.9%, respectively. The PPV for BI-RADS 5 lesions were 93.2%. The NPV for BI-RADS 3 lesions were 100%.

**Table 5:** Association between BI-RADS category and histopathological diagnosis (n = 423).

BI-RADS category	Frequency *n* (%)	HPE findings	
		Benign *n* (%)	Malignant *n* (%)
3	37 (8.7)	37 (100.0)	0
4a	133 (31.4)	125 (94.0)	8 (6.0)
4b	60 (14.2)	37 (61.7)	23 (38.3)
4c	61 (14.4)	19 (31.1)	42 (68.9)
5	132 (31.2)	9 (6.8 )	123 (93.2)
Total	423	227 (53.7)	196 (46.3)

### DBT + FFDM versus ultrasound findings

Of the 423 lesions, 368 were identified on DBT + FFDM. 55 lesions were detected on ultrasound with normal or unremarkable DBT + FFDM findings. Of the total 196 malignant lesions, 189 were identified via DBT + FFDM, giving a PPV of 51.4%. Additional 7 (12.7%) cancers were detected via ultrasound in the patients with unremarkable DBT + FFDM findings. All the malignant lesions that were not visualized in the DBT + FFDM images were in BI-RADS density C and D.

### Characteristics of mammographic abnormality

Of the 368 lesions identified on DBT + FFDM, there were 258 masses, 99 suspicious calcifications, and 61 asymmetry. These lesions were characterized as per BI-RADS lexicon for mammography and summarized in Table 6. All architectural distortion detected were in association with suspicious mass lesion, hence were categorized under associated features (Table 7).

**Table 6:** BI-RADS mammogram lexicon associated with HPE diagnosis.

Mammographic abnormalities	Description	Benign *n* (%)	Malignant *n* (%)
Mass *n* = 258	Benign	97 (91.5)	9 (8.5)
	Suspicious	17 (11.2)	135 (88.8)
	**Shape**		
	Oval	56 (76.7)	17 (23.3)
	Round	46 (86.8)	7 (13.2)
	Irregular	12 (9.1)	120 (90.9)
	**Margin**		
	Circumscribed	92 (90.2)	10 (9.8)
	Indistinct	5 (71.4)	2 (28.6)
	Obscured	2 (15.4)	11 (84.6)
	Microlobulated	12 (27.3)	32 (72.7)
	Spiculated	3 (3.3)	89 (96.7)
	**Density**		
	Fat	1 (100)	0 (0.0)
	Low	45 (96.7)	2 (4.3)
	Equal	49 (77.8)	14 (22.2)
	High	19 ( 12.9)	128 (87.1)
Asymmetry *n* = 61		24 (39.3)	37 (60.7)
	Asymmetry	16 (48.5)	17 (51.5)
	Focal	0 (0.0)	10 (100)
	Global	7 (41.2)	10 (58.8)
	Developing	1 (100)	0 (0.0)
Suspicious calcification n = 99		38 (38.4)	61 (61.1)

**Table 7:** Associated features in 189 lesions.

Associated features (*n*)	Benign *n* (%)	Malignant *n* (%)	% In total cancer (n = 189)
Architectural distortion (69)	5 (7.2)	64 (92.8)	36.5
Nipple retraction (31)	2 (6.5)	29 (93.5)	16.4
Skin thickening (62)	5 (8.1)	57 (91.9)	32.8
Skin retraction (1)	0 (0.0)	1 (100)	< 0.1
Lymphadenopathy (47)	2 (4.3)	45 (95.7)	24.9

### Masses and HPE findings

Features that were assessed for masses include shape, margin and density. Suspicious masses were those that showed irregular shape, high density and/or spiculated/indistinct margins. Some masses demonstrated benign appearance on DBT + FFDM (i.e. oval/round, circumscribed margin). However, due to suspicious features on adjunct ultrasound, these masses were biopsied.

Of the 258 masses, 152 (58.9%) appeared suspicious on DBT + FFDM. 135 (88.8%) were malignant and 17(11.2%) were benign. Of the 106 (41.1%) masses which appeared benign on DBT + FFDM, 9 (8.5%) were malignant.

Out of the 144 malignant masses detected on DBT + FFDM, only 98 (68.1%) masses can be measured as the rest have ill-defined and obscured margins. The sizes of the malignant masses ranged between 0.5 and 8.9 cm with the mean size of 2.7 cm.

**Figure 2:**
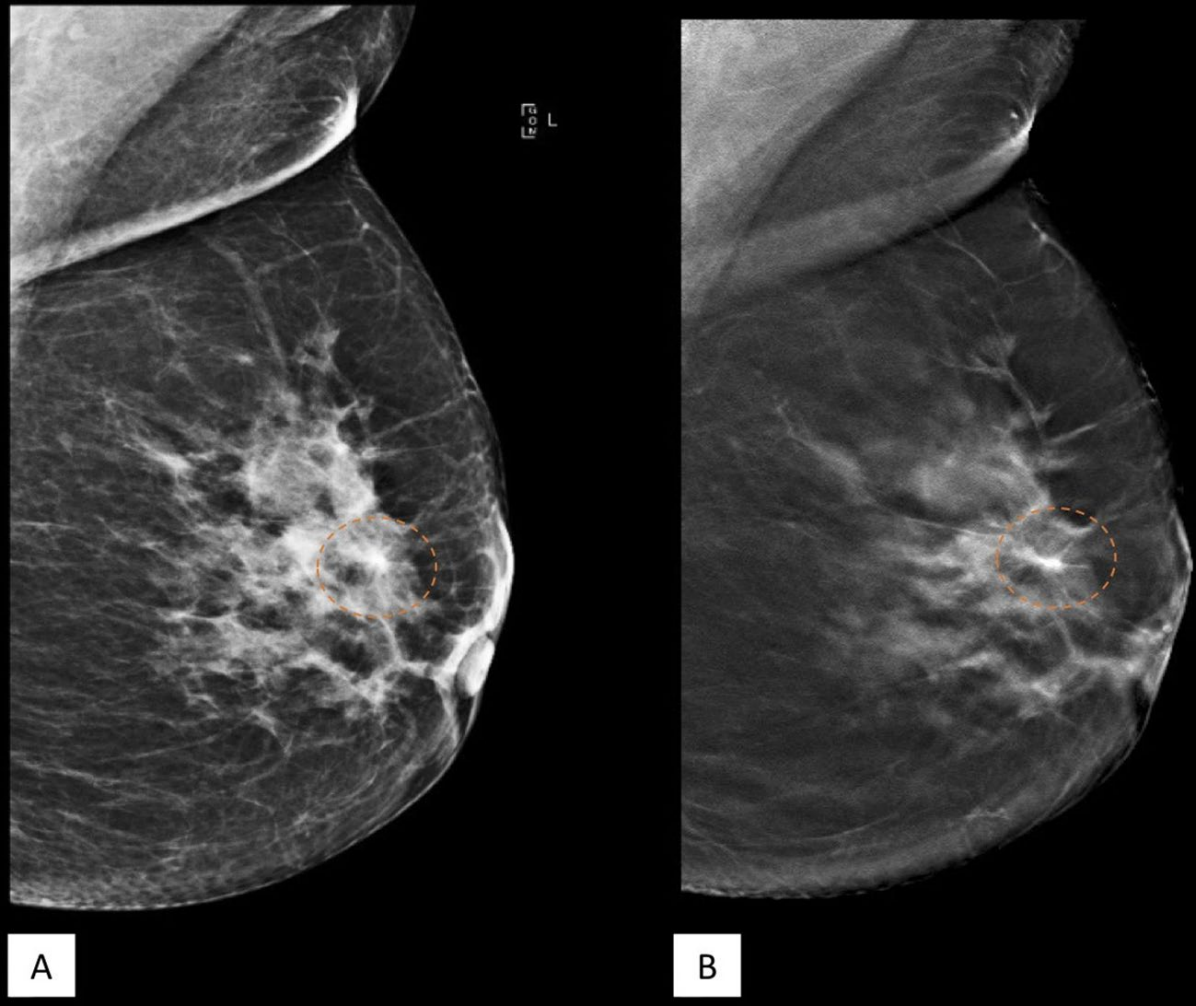
55 year-old lady who presented with left breast lump in the left breast. MLO view of the left breast in FFDM (**A**) and tomosynthesis (**B**) showing a spiculated high density lesion in the upper quadrant (dashed circle). The spiculations are pronounced in the tomosynthesis image. HPE proven DCIS.

**Figure 3:**
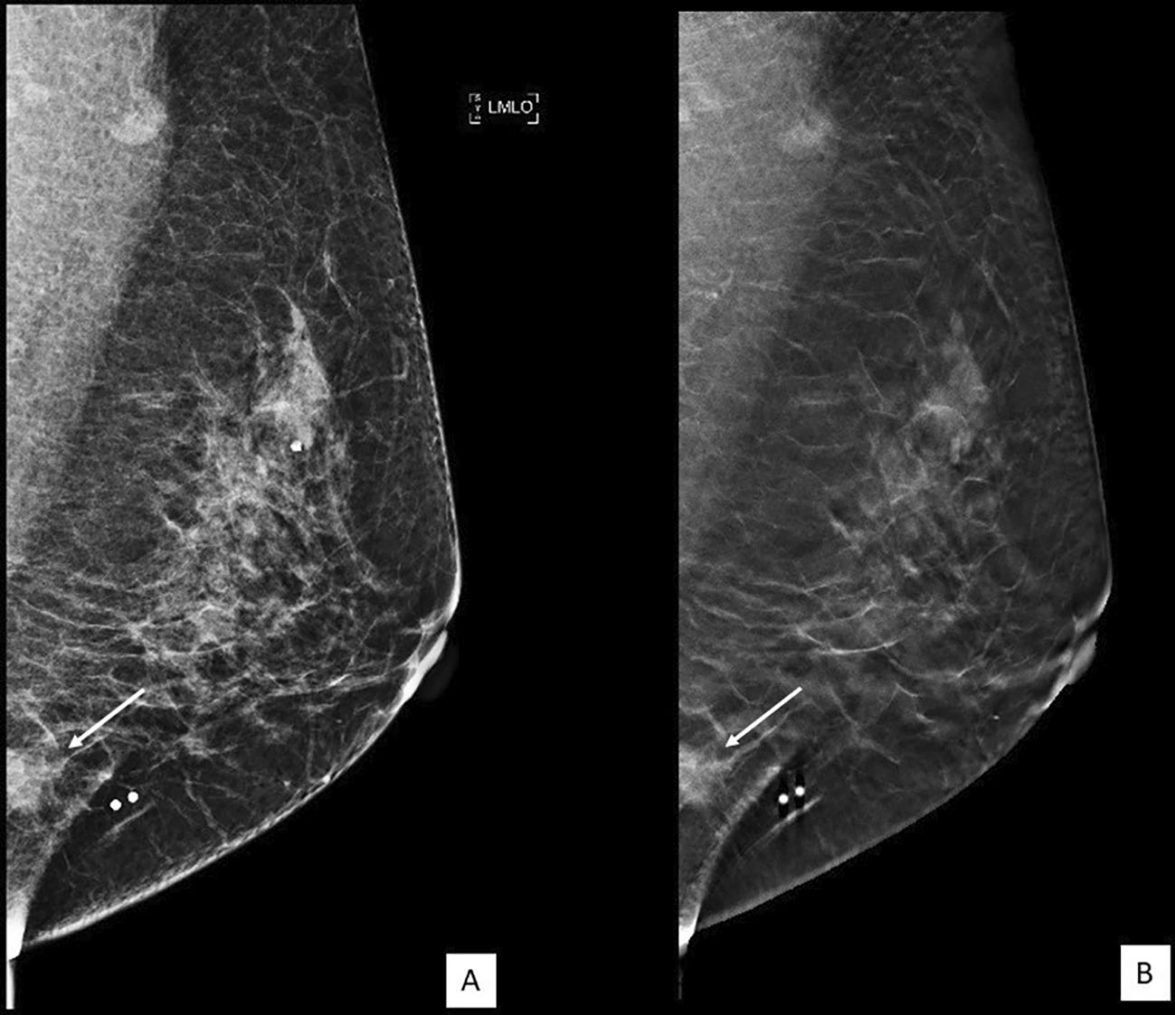
A 55 year-old lady who presented with a palpable lump. (**A**) FFDM and (**B**) tomosynthesis images of the left breast in MLO view showed an irregular high density lesion with spiculated margin in the lower quadrant on the left breast (white arrow). HPE proven invasive carcinoma.

**Figure 4:**
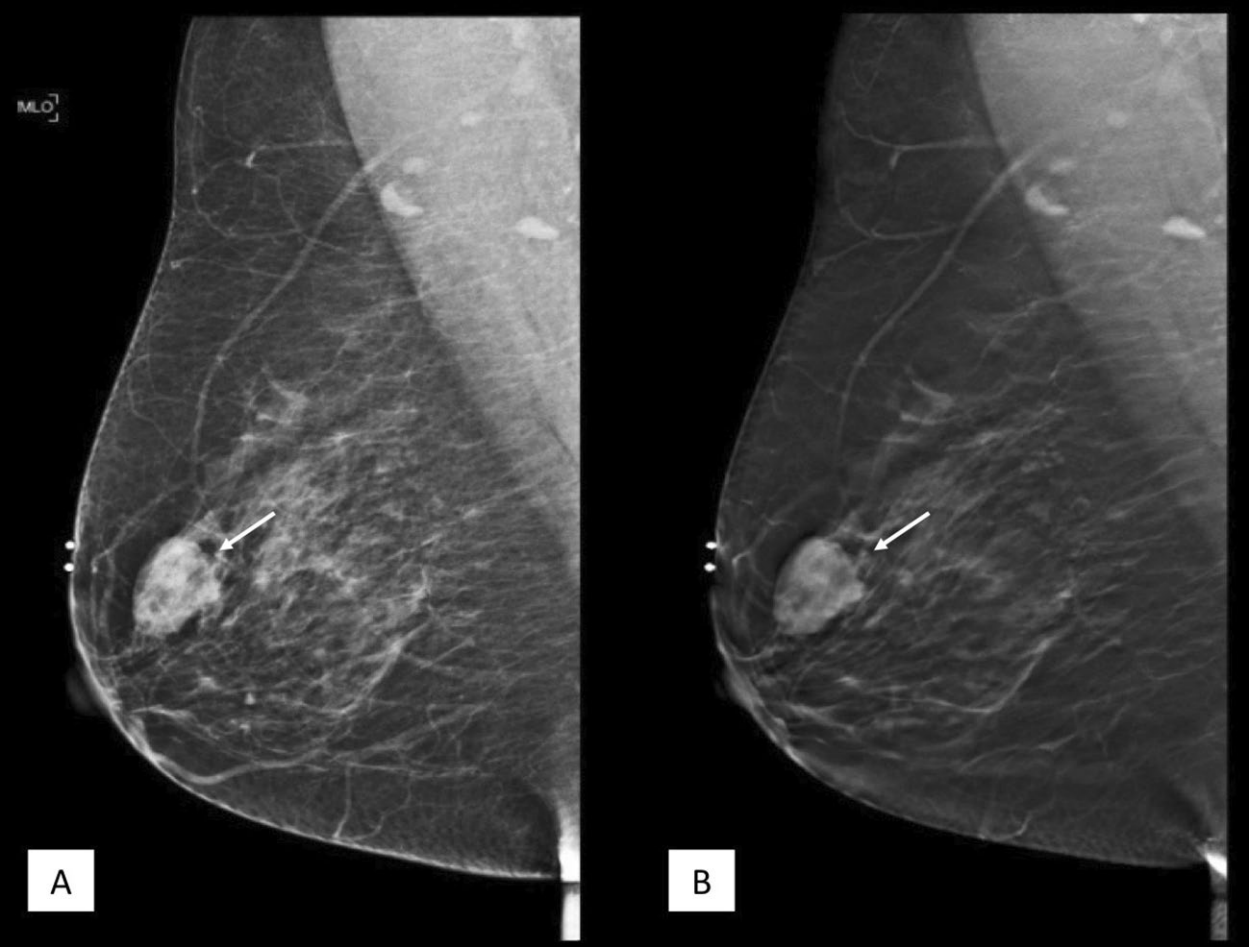
56 year-old lady who presented with right breast lump for 1 month. MLO view of the right breast in FFDM (**A**) and tomosynthesis (**B**) showing a high density lesion (white arrow) in the upper quadrant. The microlobulated margin posteriorly are clearly demarcated on tomosynthesis image. HPE proven invasive papillary carcinoma.

Figure 2, 3 and 4 are examples of spiculated lesions noted on DBT + FFDM. The spiculations are more pronounced in the DBT images. Microlobulated margin is a suspicious feature which is demonstrated in Fig. 4.

### Calcification

**Figure 5:**
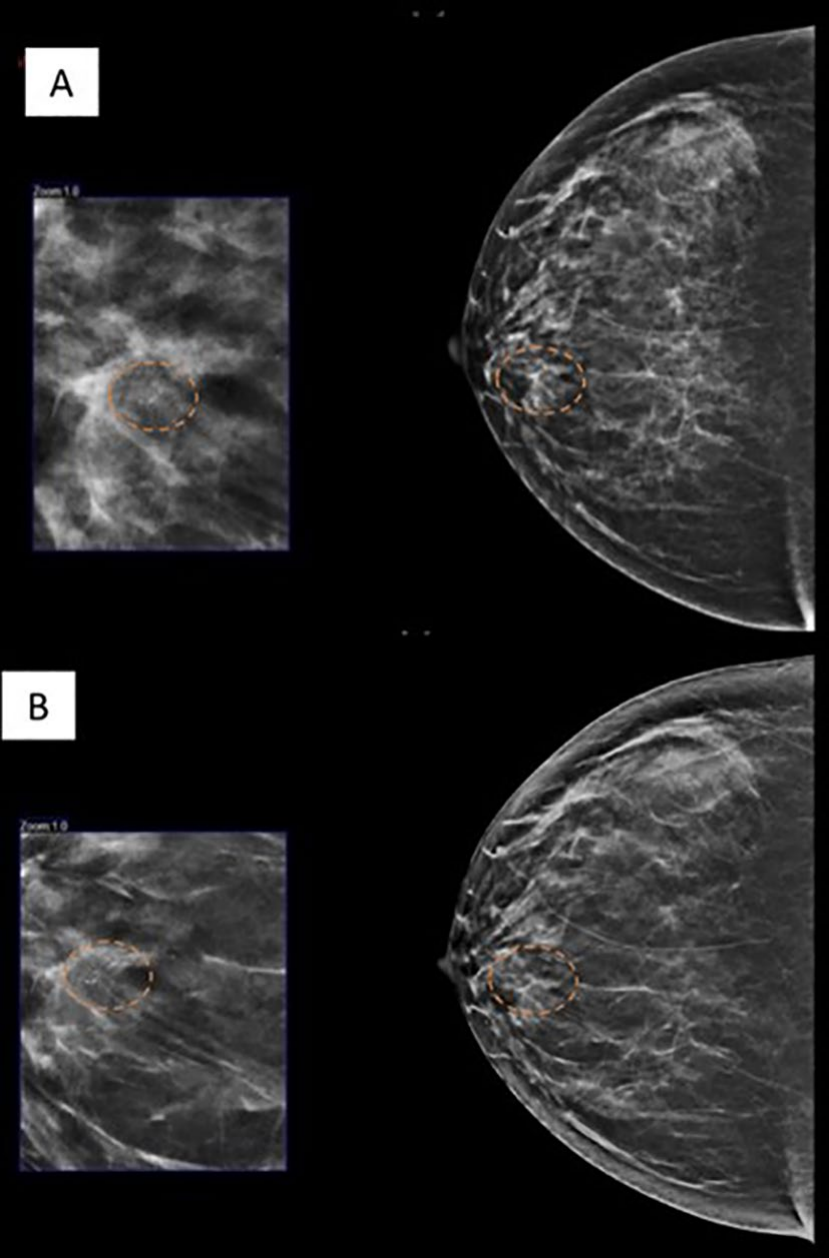
46 year-old lady who came for screening mammography. CC view of the right breast in FFDM (**A**) and C-view (C-view software by Hologic, software version 1.7, http://www.lowdose3d.com/) (**B**) showing a suspicious calcification in linear distribution in the inner quadrant (dashed circle). Vacuum assisted stereotactic guided biopsy was performed. The distribution of the calcifications were clearly seen in the synthesized 2D image from tomosynthesis. HPE proven DCIS.

There were 99 suspicious calcification; 47 (47.5%) were calcification with no associated mass and 52 (52.5%) were associated with a suspicious mass. There were 61 (61.1%) malignant lesions with PPV of 61.6%. Figure 5 showed an example of suspicious calcifications which was proven to be DCIS.

### Asymmetric density

There were 61 asymmetries detected; 37 (60.7%) were malignant and the PPV was 60.7%. The detailed description in Table 6.

### Associated features

Associated features were seen in 189 lesions. The most frequently noted feature associated with malignancy is architectural distortion (92.8%). Presence of nipple retraction, skin thickening and lymphadenopathy were also predictive of malignancy (Table 7).

### Architectural distortion

All architectural distortion detected in this cohort were associated with either a suspicious mass or calcifications.

There were 69 cases of architectural distortion reported. 92.8% (n = 64) were seen in association with a malignant lesion and 7.2% (n = 5) were in benign lesion. The PPV was 92.8%.

### Diagnostic accuracy of DBT + FFDM

The PPV, NPV, sensitivity and specificity for characteristics of masses detected on DBT + FFDM are tabulated in Table 8. The PPV for suspicious calcification was 61.62% and for asymmetric densities was 60.7%.

**Table 8:** PPV, NPV, sensitivity and specificity for mass lesion detected on DBT + FDM.

	PPV	NPV	Sensitivity	Specificity
Mass	88.82%	91.51%	93.75%	85.09%

The lesions that were deemed to be benign were followed up for 2 years and no interval cancers detected from this cohort.

#### Radiation dose

The average glandular dose (AGD) for FFDM + DBT and DBT was 4.1 mGy (95% CI 3.98–4.25) and 2.0 mGy (95% CI 1.91–2.02) respectively.

## Discussion

A number of studies have compared the clinical performance of DBT and FFDM, and have concluded that DBT showed superior image quality compared to FFDM in characterizing abnormalities and thereby more efficient in cancer detection and reduction in recall rates^9–11^.

There were 62.3% (n = 243) cases from the diagnostic group, 28.2% (n = 110) were from the opportunistic screening group and only 9.5% (n = 37) from the targeted screening group. From the 46.7% (n = 182) patients diagnosed with cancer in this study cohort, 84.6% (n = 154) were in the diagnostic group. Invasive cancers were detected in 37.8% (n = 160/423) cases and in-situ cancers were in 6.4% (n = 27/423) of cases. This is because there is no population-based breast screening programme in Malaysia, a middle-income country. Hence, most mammography cases were referrals for investigation of a symptom. Previous publication supported opportunistic screening in such setting and reported comparable performance indicators with international standards^12^. However, waiting until symptoms developed may lead to advanced cancer at presentation.

In the 187 malignant lesions, PPV in BI-RADS 4 category was 32.2%, with 4a, 4b and 4c accounted for 6.0%, 38.3% and 68.9% respectively. These are concordance with the percentage of likelihood of cancer as per ACR BI-RADS 2013 guideline; 2 to 10% for BI-RADS 4a, 10 to 50% for BI-RADS 4b and 50 to 95% for BI-RADS 4c. The PPV for BI-RADS category 5 was 93.1%. There were 9 patients with false positives in BI-RADS 5 category and this is justified as the tissue diagnosis were sclerosing adenosis, fat necrosis, and inflammation, all of which can mimic malignancies.

There were 8.7% (n = 37) in BI-RADS category 3 and of these, 78.4% (n = 29) were biopsied and proven to be non-malignant. These lesions typically demonstrate benign appearances on imaging and are routinely followed up closely on ultrasound or mammography. However, biopsy were performed in these cases to concerning risk factors or upon patient/surgeon’s request. As per the ACR BI-RADS 2013 guidelines, the likelihood of cancer of BI-RADS category 3 is 0–2%, which is in agreement with our finding.

In our study, 87.2% (n = 340) patients had mammographic abnormalities and 12.8% (n = 50) had unremarkable mammogram, but with sonographic abnormalities. There were 14.0% (n = 7/50) malignant lesions which were detected on ultrasound, but not visualized on DBT + FFDM. They were all in BI-RADS density C and D category. Therefore, supplementary ultrasound has increased our cancer detection rate by 4%. Several studies have also shown improved detection of breast cancer when ultrasound was used an adjust tool to mammography^13,14^. Corsetti et al.^13^ found that ultrasonography screening in mammography-negative patients with dense breast contributed additional 20% cancer detection rate compared to mammography alone, predominantly in women younger than 50 year-old. McCavert et al.^14^ has shown that adding ultrasonography to mammography significantly increase sensitivity in cancer detection compared to mammography alone (80.8% vs 56.6%) with no significant difference in specificity.

From the 189 malignancy cases that were identified on DBT + FFDM, 76% presented as masses, 4% as calcifications, and 20% as asymmetric density. Architectural distortion when present were all associated with either a mass, suspicious calcification or asymmetric density. The PPV for screen detected cancer in out study was 51.4%. This is in concordance with a study by Svahn et al.^15^ on comparison of diagnostic accuracy of DBT and FFDM which showed similar results, with PPV of 48.1% for DBT.

In tomosynthesis, margins of the mass lesions are delineated clearly and it was especially true for suspicious features such as indistinct, obscured and spiculated margins. In the present study, out of 149 patients with lesions that had margins with suspicious feature, 88.6% (n = 132) patients had cancer. Out of the 144 malignant masses which were mammographically detected, 89 (PPV = 96.7%) lesions were with spiculated margin, 32 (PPV = 72.7%) with indistinct margin, and 11 (PPV = 84.5%) with microlobulated margins. Only 9 (6.25%) lesions with well circumscribed margins were diagnosed to be malignant lesions.

There were 90.9% (n = 120/132) irregular shaped lesions and 87.1% (n = 128/147) high density lesions resulted positive for malignancy. 88.8% (n = 135/152) of masses that were described as suspicious were HPE proven carcinomas. One of the studies done in assessment of non-calcified breast lesions on tomosynthesis had concluded that radiologists were able to evaluate lesions more thoroughly by partially removing the tissues that are present above and below the plane of the lesion, which are overlying structures with conventional mammography, so that the shape and margin of lesions can be more readily assessed^7^. Our results were also comparable to Gilbert et al.^16^ which showed that, in cases where the dominant radiologic feature was a mass, sensitivity of DBT + FFDM was 92%. Out of the 144 masses which were malignant, 23.1% of cancers were detected in BI-RADS density A, 45.5% in BI-RADS density B, 25.9% in BI-RADS density C and 5.6% in BI-RADS density Bernardi et al.^17^ have shown that a greater advantage of tomosynthesis was observed in younger women who had dense breasts.

We described asymmetric density as asymmetry (when visualized in only one view), focal asymmetry (visualized in two views), global and developing asymmetry as per the BI-RADS lexicon for mammography. Cumulatively, 61 breast asymmetric densities were identified and biopsied and 37 (60.7%) lesions were proven to be malignant. The percentage of cancer diagnosed as asymmetry (1 view), focal asymmetry and global asymmetry were 51.1% and 58.8% respectively. Poplack et al.^10^ had also concluded similar findings to ours, whereby superior image quality were noted in the tomosynthesis images, especially in the characterization of masses and asymmetry.

Calcifications are also the commonly appearing abnormalities in mammography. In this study, C-view images were also reviewed in the characterization of suspicious breast lesions. In detection of suspicious calcifications, the PPV was 61.6%. STORM II study showed higher cancer detection rate using C-View + DBT^17^. The Oslo study as reported by Skaane et al.^18^ had concluded that application of C-view 2D to replace FFDM 2D in a tomosynthesis screening exam offered comparable clinical performance to the FFDM 2D + DBT imaging. However, a meta-analysis by Gilbert et al.^16^ has shown mixed reports in previous studies regarding detection of calcifications on DBT. The TOMMY trial has demonstrated lower specificity for calcifications than for soft tissue masses which was 88% versus 92%, respectively^19^. Detection of suspicious calcifications on mammography is important in identifying the presence of breast lesions, especially in cases of non-palpable lesions. Calcifications were clearly seen in the C-view images which were noted in previous study from our centre and also resulted in reduced radiation dose^20^. This may be due to the manufacturers’ intention to highlight calcifications and avoid the ‘thin slice’ effect of thin tomosynthesis slices^17^.

From this study, 210 lesions were found to have other associated features. Architectural distortion were commonly seen in association to malignant lesions 64 (92.8%). Other associated features, such as nipple retraction (93.5%, n = 31), lymphadenopathy (95.7%, n = 47) and skin thickening (91.9%, n = 62) were also strongly associated with cancers. In this study cohort, no architectural distortion were noted without a suspicious mass lesion. This is in contrast to other studies which noted more architectural distortion without associated mass in DBT compared to FFDM, although they were less likely to represent malignancy^21^. This may be because the readers in our study reviewed FFDM and DBT images together. More studies are recommended to assess this.

The majority of our patient population were from the Malay ethnic group. This is likely because this is the largest ethnic group in Malaysia. The cancer detection rate was similar in all other ethnic groups, which were 45.8% in Malays, 45.7% in Chinese and 50.5% in Indians. This may not be an accurate representation of the prevalence of cancer in Malaysian population. Previous study had reported higher breast cancer cases in Chinese ethnicity in Malaysia^22,23^. The patients included in this study were representative only of the cohort that attended the breast imaging unit in a tertiary referral centre in a semi-urban to urban population.

Invasive ductal carcinoma is the most common cancer across all ethnicity and age groups. The number of patients who are of less than 50 years old detected with cancer were 24% (n = 44). This is discordant with previous study on breast cancer in Asian women, which reported 40% of breast cancers diagnosed were in women aged below 50 years^24^.

The limitation of this study is it was performed in a single institution of a tertiary breast centre involving a small cohort of patients. Studies from other centres are recommended to validate our findings.

## Conclusion

We concluded that DBT is an effective tool in the diagnosis of breast cancer from a local Asian population perspective and supports the current body of literature that states that integrating DBT to FFDM allows good characterization of breast lesions and accurate diagnosis of cancer. This is supported by the diagnostic accuracy of suspicious and highly suspicious lesions which had given PPV for BI-RADS category 4 and 5 of 32.6% and 93.1%, respectively. DBT has proven to be a good diagnostic tool in identifying focal asymmetry with PPV of 100%. In addition, ultrasound has proved to be an effective adjunct tool to DBT + FFDM which yielded an additional 4% of malignancy.
